# Anorexia nervosa and Wernicke-Korsakoff syndrome: case report an literature review

**DOI:** 10.1192/j.eurpsy.2023.911

**Published:** 2023-07-19

**Authors:** S. Yelmo-Cruz, J. J. Tascon-Cervera, I. Perez-Sagaseta, C. Cardenes-Moreno, L. Torres-Tejera, A. Crisostomo-Siverio, E. Diaz-Mesa, J. Dorta-Gonzalez, M. Paniagua-Gonzalez, S. Canessa, A. L. Morera-Fumero, M. R. Cejas-Mendez

**Affiliations:** 1 Psychiatry, Hospital Universitario de Canarias; 2 Medicina interna, Dermatologia y Psiquiatría, Universidad de La Laguna, La Laguna, Spain

## Abstract

**Introduction:**

Wenicke-Korsakoff syndrome (WKS) is a neurological disorder caused by thiamine deficiency. Wernicke Encephalopathy (WE) is the acute phase and the chronic phase is called Korsakoff-syndrome (KS).

**Objectives:**

To review the current literature on the management of WKS in a patient with anorexia nervosa.

**Methods:**

We report the case of a 63-year-old woman admitted to the Psychiatry Unit after weight loss in the last 3 months (from 39 kg to 33,500 kg). She only made one meal a day. By exploration and analysis, neoplastic disease is ruled out (thoraco-abdomino-pelvic CT without pathological findings). She has maintained restrictive intakes for more than 30 years. A long-term anorexia nervosa (AN) is suspected, with a worsening of restrictive behavior in recent months. Upon admission, she has a weight of 33,500 kg and a BMI of 14,10. She has a left palpebral ptosis and an alteration of the anterograde memory as well as affectation of executive functions. Progressive oral diet is started, and due to the suspicion of a WKS, thiamine ev is started for a week and then continued with oral thiamine. Thiamine levels are extracted once the ev treatment has begun, so we do not have previous levels to know if they were decreased. Brain MRI shows bilateral hyperintensities in white matter and at supratentorial level in T2 and FLAIR. After a month and a half of admission, the patient has progressively regained weight, has managed to make adequate intakes and has improvement in memory.

**Results:**

An adverse consequence of severe malnutrition in AN due to severe food restriction and purging behavior is thiamine deficiency, and also global cerebral atrophy and concomitant cognitive deficits can be found. Thiamine deficiency occurs in 38% of individuals with AN and is often unrecognized. WKS is caused by thiamine deficiency, and WE is the acute phase of this syndrome (presentation of triad can vary). The chronic phase is KS and consists in amnesia with confabulations. WKS typically develops after malnourishment in alcoholic patients but can be associated in nonalcoholic such as prolonged intravenous feeding, hyperemesis, anorexia nervosa, refeeding after starvation, thyrotoxicosis, malabsorption syndromes; hemodialysis; peritoneal dialysis; AIDS; malignancy. WKS is a clinical diagnosis, and no specific abnormalities have been found in cerebrospinal fluid, brain imaging or electroencephalograms. MRI has a sensitivity of 53%, but high specificity of 93%, and shows an increased signal in T2 and FLAIR sequences, bilaterally symmetrical in the paraventricular regions of the thalamus, the hypothalamus, mamillary bodies, the periaquedutal region, the floor of the fourth ventricle and midline cerebellum.

**Conclusions:**

If the disorder is suspected, thiamine should be initiated immediately in order to prevent irreversible brain damage, with an estimated mortality rate of about 20%, or to the chronic form of the WE in up to 85% of survivors

**Disclosure of Interest:**

None Declared

